# Adamantane-Isothiourea Hybrid Derivatives: Synthesis, Characterization, In Vitro Antimicrobial, and In Vivo Hypoglycemic Activities

**DOI:** 10.3390/molecules22050710

**Published:** 2017-04-29

**Authors:** Lamya H. Al-Wahaibi, Hanan M. Hassan, Amal M. Abo-Kamar, Hazem A. Ghabbour, Ali A. El-Emam

**Affiliations:** 1 Department of Chemistry, College of Sciences, Princess Nourah Bint Abdulrahman University, Riyadh 11671, Saudi Arabia; lamya-alwahaibi@hotmail.com; 2 Department of Pharmaceutical Sciences, College of Pharmacy, Princess Nourah Bint Abdulrahman University, Riyadh 11671, Saudi Arabia; hananhafila@hotmail.com (H.M.H.); amabokamer@pnu.edu.sa (A.M.A.-K.); 3 Department of Microbiology, Faculty of Pharmacy, Tanta University, Tanta, Egypt; 4 Department of Pharmaceutical Chemistry, College of Pharmacy, King Saud University, Riyadh 11671, Saudi Arabia; ghabbourh@yahoo.com

**Keywords:** synthesis, adamantane, isothiourea, carbothioimidate, antimicrobial activity, hypoglycaemic activity

## Abstract

A new series of adamantane-isothiourea hybrid derivatives, namely 4-arylmethyl (*Z*)-*N*′-(adamantan-1-yl)-morpholine-4-carbothioimidates **7a**–**e** and 4-arylmethyl (*Z*)-*N*′-(adamantan-1-yl)-4-phenylpiperazine-1-carbothioimidates **8a**–**e** were prepared via the reaction of *N*-(adamantan-1-yl)morpholine-4-carbothioamide **5** and *N*-(adamantan-1-yl)-4-phenylpiperazine-1-carbothioamide **6** with benzyl or substituted benzyl bromides, in acetone, in the presence of anhydrous potassium carbonate. The structures of the synthesized compounds were confirmed by ^1^H-NMR, ^13^C-NMR, electrospray ionization mass spectral (ESI-MS) data, and X-ray crystallographic data. The in vitro antimicrobial activity of the new compounds was determined against certain standard strains of pathogenic bacteria and the yeast-like pathogenic fungus *Candida albicans*. Compounds **7b**, **7d** and **7e** displayed potent broad-spectrum antibacterial activity, while compounds **7a**, **7c**, **8b**, **8d** and **8e** were active against the tested Gram-positive bacteria. The in vivo oral hypoglycemic activity of the new compounds was carried on streptozotocin (STZ)-induced diabetic rats. Compounds **7a**, **8ab**, and **8b** produced potent dose-independent reduction of serum glucose levels, compared to the potent hypoglycemic drug gliclazide.

## 1. Introduction

**Figure 1:**
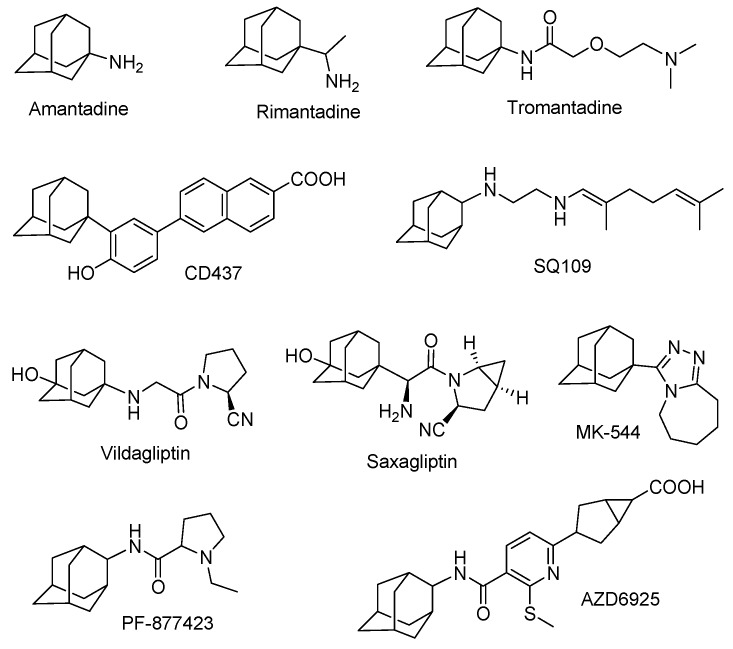
Biologically-active adamantane-based derivatives.

The adamantane nucleus was recognized early as an essential pharmacophore in various pharmacologically-active drugs. The incorporation of an adamantyl moiety into various bioactive molecules results in compounds with relatively high lipophilicity which, in turn, modifies the bioavailability and modulates their therapeutic efficacies [1,2]. Amantadine, the first adamantane-based drug, was approved for the treatment of Influenza A infection [3,4,5] and as an anti-Parkinsonian drug [6]. Further studies based on amantadine resulted in the development of the potent antiviral drugs rimantadine [7] and tromantadine [8]. Numerous adamantane-based analogues were proved to possess significant inhibitory activity against human immunodeficiency viruses (HIV) [9,10,11,12]. The synthetic retinoid derivative CD437 was developed as a potent inducer of apoptosis in human head and neck squamous cell carcinoma [13]. Potent bactericidal and fungicidal activities were reported for several adamantane derivatives, including SQ109, which was approved for use against drug-susceptible and drug-resistant TB strains [14]. SQ109 also showed excellent inhibitory activity against *Helicobacter pylori*-related duodenal ulcers and carcinomas, and *Candida glabrata* [15]. The adamantane-based drugs, vildagliptin [16], and saxagliptin [17] are currently used as oral hypoglycemic agents for the treatment of type 2 diabetes acting via inhibition of dipeptidyl peptidase IV (DPP-IV). Moreover, the adamantane derivatives MK-544 [18], PF-877423 [19], and AZD6925 [20] were recently developed as 11β-hydroxysteroid dehydrogenase type 1 (11β-HSD1) inhibitors as drug candidates for the treatment of non-insulin-dependent diabetes and obesity [21] (Figure 1).

On the other hand, several compounds containing an isothiourea moiety were reported to possess significant antiviral [22], histamine-H_3_ antagonistic [23,24], calcium channel antagonistic [25], anticancer [26], antibacterial [27], and nitric oxide synthase inhibitory [28,29] activities.

In view of the diverse pharmacological properties of adamantane and isothiourea derivatives, and following our previous studies on the chemical and biological properties of adamantane derivatives [10,30,31,32,33], we report herein the synthesis and characterization of novel adamantane derivatives containing an isothiourea moiety as potential antimicrobial and/or hypoglycemic agents.

## 2. Results and Discussion

### 2.1. Chemical Synthesis

**Scheme 1:**
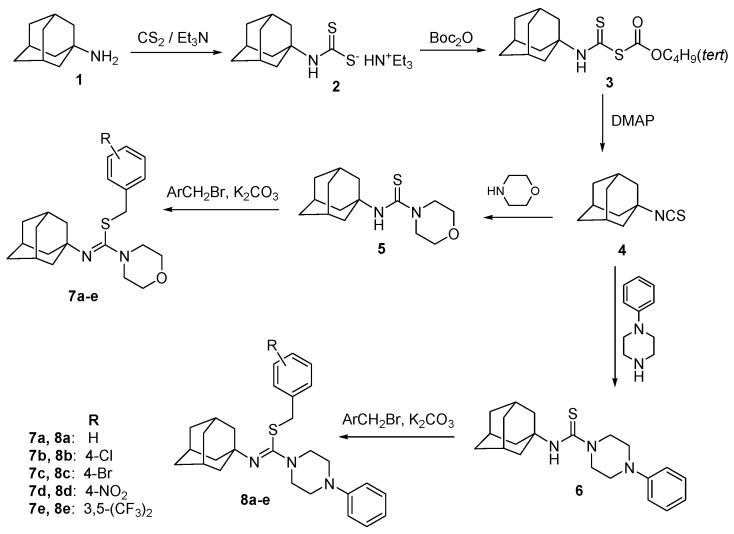
Synthetic approach for the target compounds **7a**–**e** and **8a**–**e**.

The key starting material 1-adamantyl isothiocyanate **4** was prepared in good yield via our previously described methods [33]. The reaction of 1-adamantylamine **1** with carbon disulphide and trimethylamine, in ethanol, yielded the dithiocarbamate salt **2**, which was reacted with di-*tert*-butyl dicarbonate (Boc_2_O) to yield the intermediate **3**. The intermediate **3** was stirred with catalytic amount of 4-dimethylaminopyridine (DMAP) to furnish the target product **4**. The reaction of 1-adamantyl isothiocyanate **4** with morpholine and 1-phenylpiperazine, in boiling ethanol, yielded the corresponding *N*-(adamantan-1-yl)morpholine-4-carbothioamide **5** and *N*-(adamantan-1-yl)-4-phenylpiperazine-1-carbothioamide **6**, respectively [33]. The carbothioamides **5** and **6** were reacted with benzyl or substituted benzyl bromides, in acetone, in the presence of anhydrous potassium carbonate to yield the corresponding *S*-arylmethyl derivatives **7a**–**e** and **8a**–**e**, respectively, in good yields (Scheme 1, Table 1). The structures of the target compounds **7a**–**e** and **8a**–**e** were confirmed by elemental analyses (Table S1), in addition to the ^1^H-NMR and ^13^C-NMR, and electrospray ionization mass spectral (ESI-MS) data, which were in full agreement with their structures. The ESI-MS data showed the correct positive ions (M + H)^+^ ions for all compounds. In addition, compounds **7d** and **8d** were subjected to single crystal X-ray studies.

### 2.2. Crystallographic Studies

**Figure 2:**
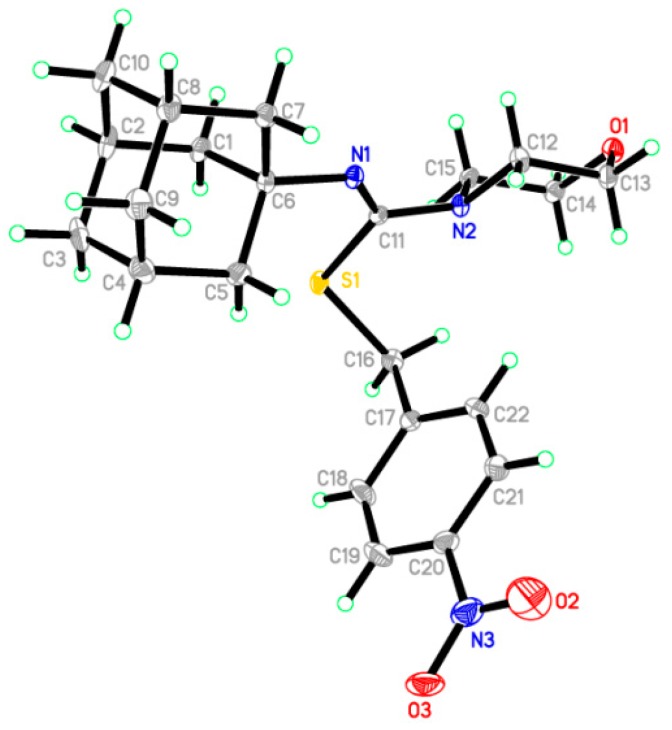
ORTEP diagram of compound **7d** drawn at 40% ellipsoids for non-hydrogen atoms.

**Figure 3:**
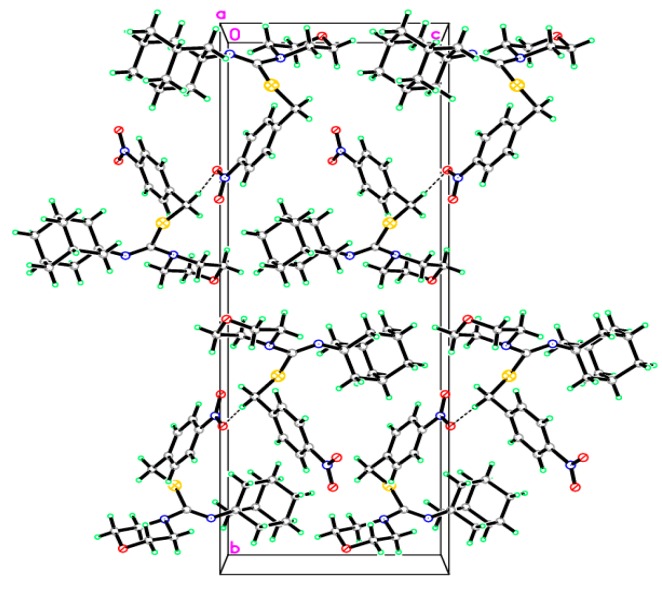
Molecular packing of compound **7d** viewed hydrogen bonds, which are drawn as dashed lines.

The single crystal X-ray crystallographic data of compounds **7d** and **8d** are summarized in Table 2. Compound **7b** crystallizes in the centrosymmetric monoclinic space group *P*2_1_/*c* with one molecule in the asymmetric unit (*Z* = 4). The ORTEP (Oak Ridge Thermal Ellipsoid Plot) is shown in Figure 2. The morpholine ring adopts a chair conformation and the mean planes of the nitrophenyl and morpholine rings make a dihedral angle of 52.55 (5). The conformation about the N1=C11 imine bond is *Z* (*cis*) configuration. The crystal packing is mainly controlled by intermolecular C-H…O hydrogen bonding (Figure 3).

**Figure 4:**
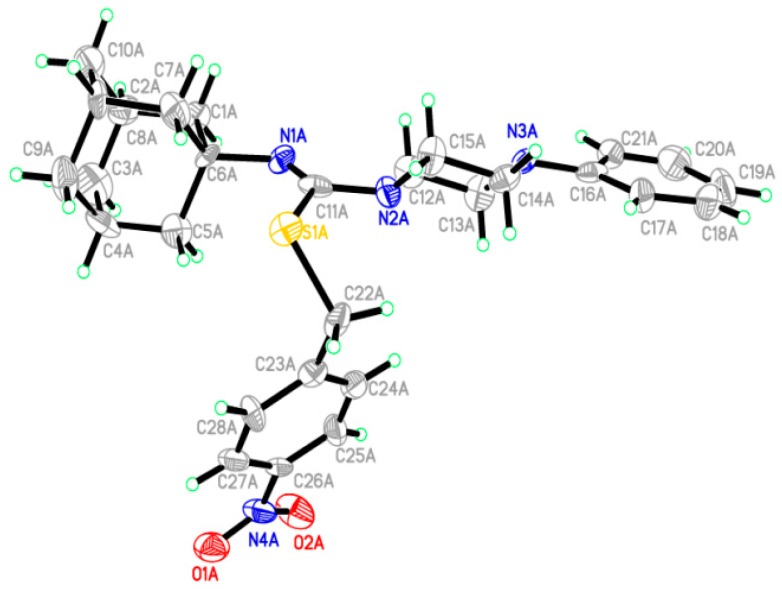
ORTEP diagram of compound **8d** drawn at 40% ellipsoids for non-hydrogen atoms.

**Figure 5:**
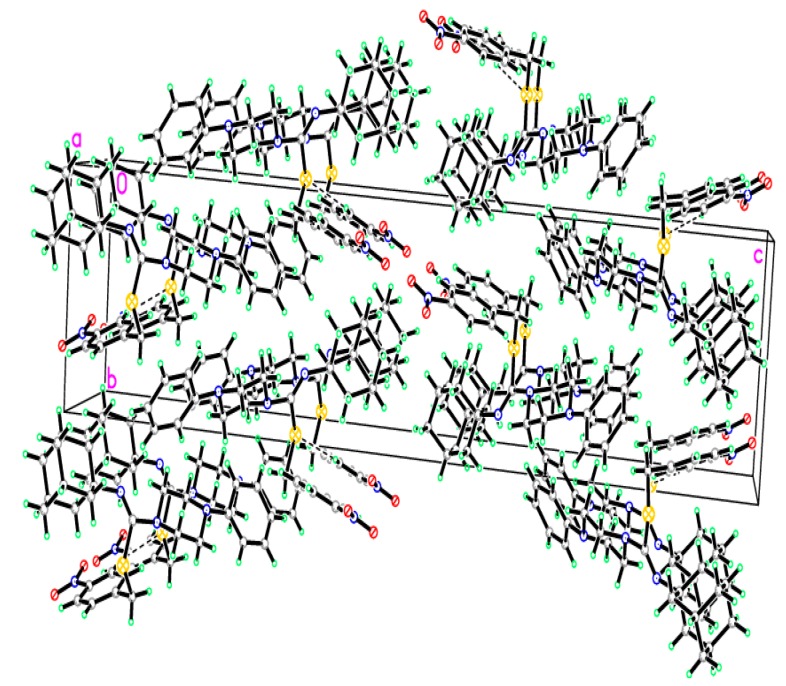
Molecular packing of compound **8d** viewed hydrogen bonds, which are drawn as dashed lines.

Compound **8b** crystallizes in the orthorhombic space group *P*2_1_2_1_2_1_ with one molecule in the asymmetric unit (*Z* = 4). The ORTEP is shown Figure 4. The piperazine ring also adopts a chair conformation with a dihedral angle of 39.25 (4). The conformation about the N1A=C11A imine bond is *Z* (*cis*) configuration. The crystal packing is mainly controlled by intermolecular C-H…S hydrogen bonding (Figure 5).

### 2.3. In Vitro Antimicrobial Activity

The newly synthesized compounds **7a**–**e** and **8a**–**e** were tested for their in vitro growth inhibitory activity against the standard bacterial strains of the American type culture collection ATCC, *Staphylococcus aureus* ATCC 6571, *Bacillus subtilis* ATCC 5256, *Micrococcus luteus* ATCC 27141 (Gram-positive bacteria), *Escherichia coli* ATCC 8726, *Pseudomonas aeruginosa* ATCC 27853 (Gram-negative bacteia), and the yeast-like pathogenic fungus *Candida albicans* MTCC 227. The preliminary antimicrobial activity testing was carried out using the semi-quantitative agar-disc diffusion method with Müller-Hinton agar medium [34]. The outcomes of the preliminary antimicrobial screening of compounds **7a**–**e**, **8a**–**e** (200 μg/disc), the antibacterial antibiotics gentamicin sulphate, ampicillin trihydrate and the antifungal drug clotrimazole (100 μg/disc) and the calculated log *P* values (Clog *P*) of the tested compounds (calculated using the CS ChemOffice Ultra version 8.0, CambridgeSoft, Cambridge, MA, USA), are shown in Table 3.

The main features of the results of the antimicrobial activity testing revealed that the tested compounds generally displayed marked antibacterial and marginal antifungal activities against the tested microorganisms, the antibacterial activity against the Gram-positive bacteria was higher than the activity against the Gram-negative bacteria, and the compound lipophilicity had no influence on their activity. In addition, the Gram-positive bacteria *S. aureus* and *B. subtilis* are more sensitive than *M. luteus*, and the Gram-negative bacteria *P. aeruginosa* was more resistant than *E. coli*. Potent antibacterial activity was shown by compounds **7a**–**e**, **8d** and **8e** which produced growth inhibition zones ≥ 20 mm against one or more of the tested microorganisms. In the morpholine derivatives **7a**–**e**, compounds **7b**, **7d** and **7e** retained good broad spectrum antibacterial activity; while compound **7a** displayed good activity against the Gram-positive bacteria and medium activity against *E. coli* (inhibition zones 15–19 mm). The optimum activity was achieved by **7e** which was highly active against all the tested bacterial strains. In the piperazine derivatives **8a**–**c**, compounds **8b**, **8d** and **8e** showed high activity against the tested Gram-positive bacteria, while compounds **8a** and **8c** showed medium activity against the tested Gram-positive bacteria and weak activity (growth inhibition zones 10–14 mm) against the tested Gram-negative bacteria. The activity against yeast-like pathogenic fungus *C. albicans* of the tested compounds was rather lower than that against the tested bacterial strains. Compound **8d** showed medium inhibitory activity, compounds **7b**, **7d**, **8a**, **8b**, **8c** and **8e** displayed weak activity and compounds **7a**, **7c** and **7e** were practically inactive (growth inhibition zones < 10 mm).

From the above results, it could be concluded that the antibacterial activity of the morpholine derivatives **7a**–**e** is generally superior to their *N*-phenylpiperazine analogues. In addition, the presence of the electron-withdrawing substituents NO_2_ and CF_3_ (compounds **7d**, **7e**, **8d** and **8e**) greatly enhanced the antibacterial activity. Unlike the antibacterial activity, the *N*-phenylpiperazine analogues **8a**–**e** were generally more active than the morpholine analogues **7a**–**e** against *C. albicans*. The values of the minimal inhibitory concentration (MIC) in Müller-Hinton Broth [35] for the tested compounds (Table 3) were correlated to the results obtained in the preliminary screening.

### 2.4. In Vivo Hypoglycemic Activity

The oral hypoglycaemic activity of compounds **7a**–**c** and **8a**–**c** was determined in streptozotocin (STZ)-induced diabetic rats. STZ induces its hyperglycemic activity via irreversible damage to the pancreatic beta cells, resulting in loss of insulin secretion [36,37]. The compounds were tried in 10 and 20 mg/kg dose levels. The dose levels (10 and 20 mg/kg) were determined after pilot experiments which showed that increasing the dose to 30 mg/kg was found to produce toxic central nervous manifestations in the form of severe symmetric convulsions. The hypoglycemic activity testing experiment (animal treatments, induction of experimental diabetes, and measurement of serum glucose level) was carried out following the previously reported protocols [32,33]. The results of the oral hypoglycemic activity of compounds **7a**–**c**, **8a**–**c** (10 and 20 mg/kg), and the potent hypoglycemic drug gliclazide in STZ-induced diabetic rats (10 mg/kg) are shown in Table 4.

The optimum hypoglycemic activity was attained by compounds **7a**, **8a**, and **8b** which produced potent dose-independent reduction of serum glucose levels, compared to gliclazide at 10 mg/kg dose level (Potency ratio 71.08, 75.13 and 79.04%, respectively). Compound **7b** showed medium dose-dependent hypoglycemic activity and compound **8c** showed weak activity at 10 mg/kg dose level without significant increase in the activity at 20 mg/kg dose level. Despite the high activity of the bis(3,5-trifluormethyl) derivative **7e** (Potency ratio 82.32%), increasing the dose produced toxic central nervous manifestations. Similarly, compound **8e** showed toxic manifestations at 10 and 20 mg/kg doses.

The hypoglycemic activity of the tested compounds revealed that the piperazine derivatives **8a**–**e** are generally more active than their morpholine analogues **7a**–**e**. In addition, the aryl substituents greatly influenced the hypoglycemic activity and toxicity. The optimum activity was attained by the phenyl derivatives (**7a**, **8a**) and to a lesser extent the chlorophenyl derivatives (**7b**, **8b**). The bis(3,5-trifluormethyl) derivatives were generally toxic.

## 3. Materials and Methods

### 3.1. General

Melting points (°C, uncorrected) were measured in open glass capillaries using a Branstead 9100 electrothermal melting point apparatus (Thermo Fisher Scientific, Waltham, MA, USA). Nuclear magnetic resonance (NMR) spectra were obtained on a Bruker Ascend 700 NMR spectrometer (Fällanden, Switzerland) at 700.17 MHz for ^1^H and 176.08 MHz for ^13^C, using CDCl_3_ as the solvent. The chemical shifts are expressed in δ (ppm) downfield from tetramethylsilane (TMS) as internal standard, coupling constants (*J*) are expressed in Hz. Electrospray ionization mass spectra (ESI-MS) were recorded on an Agilent 6410 Triple Quad tandem mass spectrometer (Agilent Technologies, Santa Clara, CA, USA) at 4.0 kV for positive ions. Elemental analyses (C, H, N, and S) were in agreement with the proposed structures within ±0.4% of the theoretical values (Table S1). Monitoring the reactions and checking the purity of the final products were carried out by thin layer chromatography (TLC) using silica gel precoated aluminum sheets (60 F_254_; Merck Schuchardt, Darmstadt, Germany), and visualization with ultraviolet light (UV) at 365 and 254 nm. The reference drugs gentamicin sulfate (CAS 1405-41-0), ampicillin trihydrate (CAS 7177-48-2), clotrimazole (CAS 23593-75-1) and gliclazide (CAS 21187-98-4), and streptozotocin (CAS 18883-66-4) were purchased from Sigma-Aldrich Chemie GmbH, Taufkirchen, Germany. The Sprauge-Dawley rats were purchased from local animal house. The commercial glucose oxidase (GO) assay kit (Sigma-Aldrich Co., St. Louis, MO, USA) were used for measurement of serum glucose levels. The animal experiments for the determination of the hypoglycemic activity were performed in accordance with the guidelines provided by the Experimental Animal Laboratory (EAL) and approved by the Animal Care and Use Committee (ACUC) of the College of Pharmacy, King Saud University (Saudi Arabia). The X-ray crystallographic data of compound **8c** was recently described [38].

### 3.2. Synthesis of 4-Arylmethyl (Z)-N′-(Adamantan-1-yl)-Morpholine-4-Carbothioimidates ***7a**–**e*** and 4-Arylmethyl (Z)-N′-(Adamantan-1-yl)-4-Phenylpiperazine-1-Carbothioimidates ***8a**–**e***

The appropriate arylmethyl bromide (2 mmol) and anhydrous potassium carbonate (276 mg, 2 mmol) were added to a solution of *N*-(adamantan-1-yl)morpholine-4-carbothioamide **5** (560 mg, 2 mmol) or *N*-(adamantan-1-yl)-4-phenylpiperazine-1-carbothioamide **6** (711 mg, 2 mmol), in anhydrous acetone (15 mL), and the mixture was heated under reflux for 4 h. The solvent was then distilled off in vacuo and the resulting residues were washed with water (20 mL), dried, and crystallized from ethanol or aqueous ethanol.

*Benzyl (Z)-N′-(adamantan-1-yl)-morpholine-4-carbothioimidate* **7a**: ^1^H-NMR: δ 1.61–1.66 (m, 6H, adamantane-H), 1.85 (m, 6H, adamantane-H), 2.0 (s, 3H, adamantane-H), 3.20–3.21 (m, 4H, morpholine-H), 3.65–3.72 (m, 4H, morpholine-H), 3.90 (s, 2H, benzylic CH_2_), 7.07–7.19 (m, 5H, Ar-H). ^13^C-NMR: δ 29.02, 29.80, 35.96, 54.18 (adamantane-C), 37.06 (benzylic CH_2_), 49.66, 66.42 (morpholine-C), 125.60, 126.44, 127.90, 140.02 (Ar-C), 154.46 (C=N). ESI-MS, *m/z*: 372.3 (M + H)^+^.

*4-Chlorobenzyl (Z)-N′-(adamantan-1-yl)-morpholine-4-carbothioimidate* **7b**: ^1^H-NMR: δ 1.62–1.65 (m, 6H, adamantane-H), 1.69–1.72 (m, 6H, adamantane-H), 2.05–2.06 (m, 3H, adamantane-H), 3.99 (s, 2H, benzylic CH_2_), 3.20–3.22 (m, 4H, morpholine-H), 3.65–3.72 (m, 4H, morpholine-H), 6.99 (d, 2H, Ar-H, *J* = 7.5 Hz), 7.14 (d, 2H, Ar-H, *J* = 7.5 Hz). ^13^C-NMR: δ 29.26, 29.99, 35.98, 54.08 (adamantane-C), 36.98 (benzylic CH_2_), 48.60, 66.44 (morpholine-C), 127.65, 128.65, 133.0, 138.04 (Ar-C), 154.24 (C=N). ESI-MS, *m/z* (Rel. Int.): 405.4 (M + H, 100)^+^, 407.4 (M + 2 + H, 35)^+^.

*4-Bromobenzyl (Z)-N′-(adamantan-1-yl)-morpholine-4-carbothioimidate* **7c**: ^1^H-NMR: δ 1.63–1.69 (m, 6H, adamantane-H), 1.84 (m, 6H, adamantane-H), 2.01 (s, 3H, adamantane-H), 3.25–3.30 (m, 4H, morpholine-H), 3.69–3.74 (m, 4H, morpholine-H), 3.91 (s, 2H, benzylic CH_2_), 7.17 (d, 2H, Ar-H, *J* = 7.5 Hz), 7.45 (d, 2H, Ar-H, *J* = 7.5 Hz). ^13^C-NMR: δ 29.59, 29.94, 36.57, 54.69 (adamantane-C), 37.76 (benzylic CH_2_), 49.70, 66.85 (morpholine-C), 121.0, 130.51, 137.26, 146.91 (Ar-C), 156.46 (C=N). ESI-MS, *m/z* (Rel. Int.): 449.4 (M + 2 + H, 100)^+^, 451.4 (M + 2 + H, 98)^+^.

*4-Nitrobenzyl (Z)-N′-(adamantan-1-yl)-morpholine-4-carbothioimidate* **7d**: ^1^H-NMR: δ 1.62 (s, 6H, adamantane-H), 1.77–1.79 (m, 6H, adamantane-H), 1.98–2.0 (m, 3H, adamantane-H), 3.24–3.30 (m, 4H, morpholine-H), 3.69–3.72 (m, 4H, morpholine-H), 4.02 (s, 2H, benzylic CH_2_), 7.42 (d, 2H, Ar-H, *J* = 8.2 Hz), 8.21 (d, 2H, Ar-H, *J* = 8.2 Hz). ^13^C-NMR: δ 29.58, 29.84, 36.44, 54.79 (adamantane-C), 37.53 (benzylic CH_2_), 49.79, 66.78 (morpholine-C), 123.76, 129.64, 146.07, 146.91 (Ar-C), 156.71 (C=N). ESI-MS, *m/z*: 416.2 (M + H)^+^.

*3,5-Bis(trifluoromethyl)benzyl (Z)-N′-(adamantan-1-yl)-morpholine-4-carbothioimidate* **7e**: ^1^H-NMR: δ 1.53–1.56 (m, 12H, adamantane-H), 1.85–1.87 (m, 3H, adamantane-H), 3.10–3.12 (m, 4H, morpholine-H), 3.68–3.70 (m, 4H, morpholine-H), 4.17 (s, 2H, benzylic CH_2_), 7.95 (s, 1H, Ar-H), 8.0 (s, 2H, Ar-H). ^13^C-NMR: δ 29.16, 29.66, 35.25, 54.85 (adamantane-C), 36.41 (benzylic CH_2_), 49.76, 66.20 (morpholine-C), 120.76, 122.72, 130.39, 142.78 (Ar-C), 124.89 (CF_3_), 148.37 (C=N). ESI-MS, *m/z*: 507.2 (M + H)^+^.

*Benzyl (Z)-N′-(adamantan-1-yl)-4-phenylpiperazine-1-carbothioimidate* **8a**: ^1^H-NMR: δ 1.69 (s, 6H, adamantane-H), 1.74 (s, 6H, adamantane-H), 2.0 (s, 3H, adamantane-H), 2.88–2.92 (m, 4H, piperazine-H), 3.02–3.06 (m, 4H, piperazine-H), 3.98 (s, 2H, benzylic CH_2_), 6.84–7.04 (m, 5H, Ar-H), 7.12–7.16 (m, 5H, Ar-H). ^13^C-NMR: δ 29.10, 29.96, 35.68, 53.98 (adamantane-C), 46.90, 48.18 (piperazine-C), 36.90 (benzylic CH_2_), 114.28, 119.90, 126.92, 128.24, 129.0, 130.58, 139.94, 149.26 (Ar-C), 152.0 (C=N). ESI-MS, *m/z*: 446.3 (M + H)^+^.

*4-Chlorobenzyl (Z)-N′-(adamantan-1-yl)-4-phenylpiperazine-1-carbothioimidate* **8b**: ^1^H-NMR: δ 1.71–1.76 (m, 12H, adamantane-H), 2.15–2.17 (m, 3H, adamantane-H), 2.63–2.65 (m, 4H, piperazine-H), 3.22–3.24 (m, 4H, piperazine-H), 3.98 (s, 2H, benzylic CH_2_), 6.89–6.91 (m, 3H, Ar-H), 6.95–6.96 (m, 2H, Ar-H), 7.29–733 (m, 4H, Ar-H). ^13^C-NMR: δ 29.17, 29.71, 35.51, 53.07 (adamantane-C), 48.65, 48.10 (piperazine-C), 36.39 (benzylic CH_2_), 115.55, 116.13, 119.89, 128.49, 129.16, 129.45, 130.57, 150.31 (Ar-C), 151.27 (C=N). ESI-MS, *m/z* (Rel. Int.): 380.2 (M + H, 100)^+^, 382.2 (M + 2 + H, 37)^+^.

*4-Bromobenzyl (Z)-N′-(adamantan-1-yl)-4-phenylpiperazine-1-carbothioimidate* **8c**: ^1^H-NMR: δ 1.65 (s, 6H, adamantane-H), 1.86 (s, 6H, adamantane-H), 2.02–2.03 (m, 3H, adamantane-H), 3.26–3.27 (m, 4H, piperazine-H), 3.43–3.44 (m, 4H, piperazine-H), 3.95 (s, 2H, benzylic CH_2_), 6.92–7.93 (m, 1H, Ar-H), 7.00–7.01 (m, 2H, Ar-H), 7.18 (d, 2H, Ar-H, *J* = 7.0 Hz), 7.29–7.33 (m, 2H, Ar-H), 7.44 (d, 2H, Ar-H, *J* = 7.0 Hz). ^13^C-NMR: δ 29.95, 36.58, 42.98, 54.70 (adamantane-C), 48.96, 49.17 (piperazine-C), 37.77 (benzylic CH_2_), 116.20, 119.97, 120.94, 129.22, 130.59, 131.54, 137.33, 149.26 (Ar-C), 151.29 (C=N). ESI-MS, *m/z* (Rel. Int.): 524.4 (M + H, 98)^+^, 526.4 (M + 2 + H, 100)^+^ [38].

*4-Nitrobenzyl (Z)-N′-(adamantan-1-yl)-4-phenylpiperazine-1-carbothioimidate* **8d**: ^1^H-NMR: δ 1.53 (s, 6H, adamantane-H), 1.72 (s, 6H, adamantane-H), 1.91 (s, 3H, adamantane-H), 3.16–3.18 (m, 4H, piperazine-H), 3.30–3.33 (m, 4H, piperazine-H), 3.97 (s, 2H, benzylic CH_2_), 6.80–6.89 (m, 3H, Ar-H), 7.18–7.29 (m, 2H, Ar-H), 7.36 (d, 2H, Ar-H, *J* = 8.0 Hz), 8.08 (d, 2H, Ar-H, *J* = 8.0 Hz). ^13^C-NMR: δ 29.65, 29.90, 36.52, 54.86 (adamantane-C), 37.54 (benzylic CH_2_), 43.03, 49.12 (piperazine-C), 116.21, 120.05, 123.72, 129.21, 129.66, 146.14, 146.94, 148.09 (Ar-C), 151.22 (C=N). ESI-MS, *m/z*: 491.2 (M + H)^+^.

*3,5-Bis(trifluoromethyl)benzyl (Z)-N′-(adamantan-1-yl)-4-phenylpiperazine-1-carbothioimidate* **8e**: ^1^H-NMR: δ 1.58 (s, 6H, adamantane-H), 1.69 (s, 6H, adamantane-H), 1.95–1.97 (m, 3H, adamantane-H), 3.29–3.31 (m, 4H, piperazine-H), 3.40–3.42 (m, 4H, piperazine-H), 4.04 (s, 2H, benzylic CH_2_), 6.93–7.02 (m, 3H, Ar-H), 7.29 (s, 1H, Ar-H), 7.32–7.34 (m, 2H, Ar-H), 7.78 (s, 2H, Ar-H). ^13^C-NMR: δ 29.80, 35.51, 36.44, 54.80 (adamantane-C), 37.31 (benzylic CH_2_), 49.0, 49.13 (piperazine-C), 116.28, 120.12, 129.16, 129.24, 131.44, 131.63, 141.30, 147.50 (Ar-C), 124.04 (CF_3_), 151.19 (C=N). ESI-MS, *m/z*: 582.2 (M + H)^+^.

### 3.3. Crystal Growth and Single Crystal X-ray Study

Single crystals suitable for X-ray analysis were obtained by slow evaporation of CHCl_3_:EtOH (1:1; 5 mL) solution of compounds **7d** and **8d** at room temperature. Data were collected on a Bruker APEX-II D8 Venture area diffractometer, equipped with graphite monochromatic Mo *K*α radiation (λ = 0.71073 Å) at 100 K. Unit cell measurement, data collection, integration, scaling, and absorption corrections for the crystals were conducted using Bruker Apex II software [39]. Data reduction was done by Bruker SAINT suite [40]. The crystal structures were solved by the full matrix least squares method using SHELXL 2014 [41]. Absorption correction was applied using SADABS program [42]. ORTEP (Oak Ridge Thermal Ellipsoid Plot) was generated using Mercury 3.5.1 Cambridge Crystallographic Data Centre (CCDC) program [43].

## 4. Conclusions

In this study, new adamantane-linked isothiourea derivatives were synthesized and their in vitro antimicrobial and in vivo hypoglycemic activities were determined. Compounds **7b**, **7d**, and **7e** displayed potent broad-spectrum antibacterial activity, while compounds **7a**, **7c**, **8b**, **8d**, and **8e** were active against the tested Gram-positive bacteria. The tested compounds were generally inactive against the yeast-like pathogenic fungus *Candida albicans*. The in vivo oral hypoglycemic activity of the synthesized compounds was determined in streptozotocin (STZ)-induced diabetic rats. Compounds **7a**, **8a**, and **8b** produced potent dose-independent reduction of serum glucose levels compared to gliclazide at 10 mg/kg dose level (potency ratios of 71.08%, 75.13%, and 79.04%, respectively). The active compounds are considered to be good candidates as newer antibacterial and hypoglycemic agents, though, further studies including toxicity testing and molecular docking for the exploration of the mechanism of their biological activity are required for optimization of the activity which are being undertaken.

## Figures and Tables

**Table 1:** Crystallization solvents, melting points, yield percentages, molecular formulae, and molecular weights of compounds **7a**–**e** and **8a**–**e**.

Comp. No.	R	Cryst. Solv.	M.p. (°C)	Yield (%)	Mol. Formula (Mol. Wt.)
**7a**	H	EtOH/H_2_O	108–110	91	C_22_H_30_N_2_OS (370.55)
**7b**	4-Cl	EtOH	92–94	76	C_22_H_29_ClN_2_OS (405.0)
**7c**	4-Br	EtOH	98–100	85	C_22_H_29_BrN_2_OS (449.45)
**7d**	4-NO_2_	EtOH	118–120	95	C_22_H_29_N_3_O_3_S (415.55)
**7e**	3,5-(CF_3_)_2_	EtOH/H_2_O	106–108	72	C_24_H_28_F_6_N_2_OS (506.55)
**8a**	H	EtOH/H_2_O	137–139	88	C_28_H_35_N_3_S (445.66)
**8b**	4-Cl	EtOH	153–155	90	C_28_H_34_ClN_3_S (480.11)
**8c**	4-Br	EtOH	140–142	92	C_28_H_34_BrN_3_S (524.56)
**8d**	4-NO_2_	EtOH	145–147	96	C_28_H_34_N_4_O_2_S (490.66)
**8e**	3,5-(CF_3_)_2_	EtOH/H_2_O	113–115	75	C_30_H_33_F_6_N_3_S (581.66)

**Table 2:** Single-crystal X-ray crystallographic data of compounds **7d** and **8d**.

Data	Compound 7d	Compound 8d
Formula	C_22_H_29_N_3_O_3_S	C_28_H_34_N_4_O_2_S
Formula weight	415.55	490.66
Temperature (K)	293	293
Wavelength (Å)	0.71073	0.71073
Crystal system	Monoclinic	Orthorhombi
Space group	*P*2_1_/*c*	*P*2_1_2_1_2_1_
*a*, *b*, *c* (Å)	6.9204 (5), 29.775 (3), 10.2725 (10)	6.9426 (9), 9.6472 (12), 39.086 (5)
V (Å^3^)	2116.7 (3)	2617.8 (6)
*Z*	4	4
Radiation type	Mo *K*α	Mo *K*α
*μ* (mm^−1^)	0.18	0.16
No. of reflections	11033	25091
No. of unique reflections/obs. reflections	3718/2253	4609/1447
No. of parameters	262	318
No. of restraints	0	0
Δρmax, Δρmin (e Å^−3^)	0.28, −0.21	0.44, −0.40
*T*_min_, *T*_max_	0.939, 0.989	0.924, 0.957
*R*_int_	0.073	0.526
Crystal size (mm)	0.35 × 0.11 × 0.06	0.85 × 0.21 × 0.05
R[*F*^2^ > 2σ(*F*^2^)], wR(*F*^2^), S	0.052, 0.192, 0.65	0.128, 0.296, 1.02
CCDC number	1525183	1523432

**Table 3:** Antimicrobial activity of compounds **7a**–**e** and **8a**–**e** (200 μg/8 mm disc), the broad-spectrum antibacterial drugs gentamicin sulphate, ampicillin trihydrate and the antifungal drug clotrimazole (100 μg/8 mm disc) against *Staphylococcus aureus* ATCC 6571 (SA), *Bacillus subtilis* ATCC 5256 (BS), *Micrococcus luteus* ATCC 27141 (ML), *Escherichia coli* ATCC 8726 (EC), *Pseudomonas aeruginosa* ATCC 27853 (PA), and the yeast-like pathogenic fungus *Candida albicans* MTCC 227 (CA).

Comp. No.	Clog *P*	Diameter of Growth Inhibition Zone (mm) ^a^					
		SA	BS	ML	EC	PA	CA
**7a**	5.584	22 (2) ^b^	21 (4) ^b^	20 (4) ^b^	18 (16) ^b^	14 (64) ^b^	-
**7b**	6.297	24 (4) ^b^	28 (0.5) ^b^	22 (4) ^b^	22 (20) ^b^	15 (32)	11 (>128) ^b^
**7c**	6.447	22 (4) ^b^	18 (16) ^b^	14 (64) ^b^	13 (128) ^b^	12 (128) ^b^	-
**7d**	5.327	31 (0.5) ^b^	32 (0.25) ^b^	28 (0.5) ^b^	22 (1) ^b^	18 (4) ^b^	14 (32) ^b^
**7e**	7.350	33 (0.25) ^b^	34 (0.25) ^b^	28 (1) ^b^	24 (2) ^b^	20 (4) ^b^	-
**8a**	7.130	18 (8) ^b^	18 (8) ^b^	14 (128) ^b^	12 (>128) ^b^	10 (>128) ^b^	10 (>128) ^b^
**8b**	7.843	21 (8) ^b^	24 (2) ^b^	16 (32) ^b^	16 (64) ^b^	12 (>128) ^b^	13 (64) ^b^
**8c**	7.993	17 (32) ^b^	19 (8) ^b^	14 (64) ^b^	11 (>128) ^b^	10 (>128) ^b^	12 (128) ^b^
**8d**	6.873	24 (1) ^b^	28 (1) ^b^	20 (2) ^b^	18 (2) ^b^	14 (4) ^b^	16 (16) ^b^
**8e**	8.896	28 (1) ^b^	31 (0.5) ^b^	22 (2) ^b^	19 (4) ^b^	18 (8) ^b^	14 (64) ^b^
**Gentamicin sulfate**		27 (1) ^b^	26 (2) ^b^	20 (2) ^b^	22 (0.5) ^b^	21 (0.5) ^b^	NT
**Ampicillin trihydrate**		22 (2) ^b^	23 (1) ^b^	20 (2) ^b^	16 (8) ^b^	16 (8) ^b^	NT
**Clotrimazole**		NT	NT	NT	NT	NT	21 (4) ^b^

^a^ (-): inactive (inhibition zone < 10 mm), ^b^ Figures shown in parentheses represent the MIC values (μg/mL), NT: not tested.

**Table 4:** Oral hypoglycemic activity of compounds **7a**–**c**, **8a**–**c** (10 and 20 mg/kg), and gliclazide (10 mg/kg) in STZ-induced diabetic rats.

Treatment	Results		
	C_0_ (mg/dL) ^a^	C_24_ (mg/dL) ^a^	% Glucose Reduction ^b^
Group 1 ^c^	302.8 ± 11.64	290.2 ± 18.22	4.16%
Group 1 ^d^	299.2 ± 16.50	171.6 ± 12.32 *	42.65%
**7a** (10 mg/kg)	304.8 ± 13.26	212.4 ± 12.16 *	30.31% (71.08)
**7a** (20 mg/kg)	300.6 ± 11.65	134.6 ± 9.75 *	55.22% (64.74)
**7b** (10 mg/kg)	288.9 ± 12.15	245.2 ± 19.25 *	15.13% (35.47)
**7b** (20 mg/kg)	294.8 ± 9.08	201.5 ± 9.60 *	31.65% (37.13)
**7c** (10 mg/kg)	284.8 ± 19.55	281.2 ± 7.19	1.26% (2.69)
**7c** (20 mg/kg)	290.2 ± 21.64	286.8 ± 19.02	2.75% (1.37)
**7d** (10 mg/kg)	278.1 ± 16.24	282.2 ± 27.20	−1.47%
**7d** (20 mg/kg)	302.6 ± 22.25	299.8 ± 18.80	0.93% (1.08)
**7e** (10 mg/kg)	306.2 ± 15.20	198.7 ± 19.10 *	35.12 (82.32)
**7e** (20 mg/kg)	Toxic		
**8a** (10 mg/kg)	294.6 ± 11.30	200.2 ± 9.88 *	32.04% (75.13)
**8a** (20 mg/kg)	290.6 ± 8.60	108.4 ± 11.05 *	62.70% (73.50)
**8b** (10 mg/kg)	301.4 ± 9.06	199.8 ± 10.01 *	33.71% (79.04)
**8b** (20 mg/kg)	296.0 ± 11.02	144.6 ± 10.01 *	51.15% (59.96)
**8c** (10 mg/kg)	320.5 ± 22.05	277.6 ± 16.20	13.39% (31.38)
**8c** (20 mg/kg)	313.5 ± 18.60	269.9 ± 20.12	13.91% (16.30)
**8d** (10 mg/kg)	295.0 ± 22.45	289.2 ± 25.28	1.97% (4.61)
**8d** (20 mg/kg)	304.5 ± 27.50	309.0 ± 25.95	−1.48
**8e** (10 mg/kg)	286.6 ± 13.22	178.2 ± 16.04 *	37.82% (88.68)
**8e** (20 mg/kg)	Toxic		

^a^ Results are expressed as mean ± S.E.M. (*n* = 5), ^b^ The figures shown in parentheses are the relative potency compared with gliclazide, ^c^ Treated with a single oral dose of 0.5% (*w*/*v*) aqueous CMC solution (5 mL/kg), ^d^ Treated with 10 mg/kg gliclazide in 0.5% (*w*/*v*) aqueous CMC, * Significant difference at *p* < 0.01 compared with the corresponding control.

## Supplementary Materials

Supplementary materials (the experimental details of the determination of in vitro antimicrobial activity, in vivo hypoglycemic activity and microanalytical data) are available online.
