# Supplementary material for: Adamantane-Isothiourea Hybrid Derivatives: Synthesis, Characterization, In Vitro Antimicrobial, and In Vivo Hypoglycemic Activities
Source: Molecules. 2017 Apr 29;22(5):710. doi: 10.3390/molecules22050710 (PMC6154638; doi:10.3390/molecules22050710)
Supplement: Supplementary file 1 [file molecules-22-00710-s001.pdf]

Supplementary Materials

Adamantane-Isothiourea Hybrid Derivatives: Synthesis, Characterization, *In Vitro* Antimicrobial and *In Vivo* Hypoglycemic Activities

Determination of in vitro antimicrobial activity (agar disc diffusion method)

Sterile filter paper discs (8 mm diameter) were moistened with the compound solution in dimethyl sulphoxide of specific concentration (200 µg/disc), and the antibacterial drugs, gentamicin sulphate and ampicillin trihydrate (100 µg/disc) and the antifungal drug clotrimazole (100 µg/disc), were carefully placed on agar culture plates that had been previously inoculated separately with the microorganisms. The plates were incubated at 37 °C, and the diameters of the growth inhibition zones were measured after 24 hours for bacteria and 48 hours for *C. albicans*.

Determination of the minimal inhibitory concentration (MIC)

Compounds **7a-e**, **8a-e**, gentamicin sulphate, ampicillin trihydrate, and clotrimazole were dissolved in dimethyl sulphoxide at a concentration of 128 µg/mL. The two-fold dilutions of the solution were prepared (128, 64, 32, …, 0.25 µg/mL). Suspensions of the microorganisms at concentrations of 106 colony-forming units per mL were inoculated in the corresponding wells. The plates were then incubated at 36°C for 24 hours. The MIC values were determined as the lowest concentrations that completely inhibited visible growth of the microorganism as detected by the unaided eye.

Determination of the in vivo hypoglycemic activity

Animals: Locally bred male Sprauge-Dawley rats (250 ± 30 g body weight) were housed in wire-bottomed cages at 22±2 °C. A standard pellet diet and tap water were supplied *ad libitium*. The animals were acclimatized to these conditions for 15 days before the experiment.

Induction of experimental diabetes: The animals were fasted for 16 hours before the induction of diabetes with STZ. The animals were injected intraperitoneally with 0.22-0.25 mL of a freshly prepared solution STZ (60 mg/mL in 0.01 *M* citrate buffer, pH 4.5) at a final dose of 60 mg/kg body weight. Only rats with serum glucose levels greater than 250 mg/dL were used in experiments.

Design of the experiment: Homogenous suspensions of compounds **7a-e**, **8a-e** and the oral hypoglycaemic drug gliclazide in 0.5% (w/v) aqueous carboxymethyl cellulose (CMC) solution

were prepared at specific concentration of 10 mg/mL. The hypoglycemic activity of the compounds **7a-e**, **8a-e** was assessed 48 hours post STZ injection. The diabetic rats were fasted for 16 hours and divided into 18 groups each of 5 animals (n = 5) and the serum glucose level was determined for each group and considered as the initial fasting serum glucose (C_0). Group 1, which served as the negative diabetic control group, received a single oral dose of 0.5% (w/v) aqueous CMC solution (5 mL/kg). Groups 2 was treated with 10 mg/kg gliclazide in 0.5% (w/v) aqueous CMC (positive control). Groups 3-18 were treated with either a single oral dose of the 10 or 20 mg/kg of the test compounds. All treatments were administered by oral gavage. 24 Hours after treatment, the blood samples were collected and the serum glucose level (C_{24}) was determined for each group.

Determination of serum glucose: Blood samples from the animal tail vein were collected, allowed to clot, centrifuged at 2000 r.p.m. for 10 minutes. Blood glucose levels were expressed in mg/dL as mean ± SEM. The data were statistically analysed using ANOVA with Tukey's multiple comparison test. The values of $p < 0.01$ were considered as significant. The percentage of serum glucose reduction for each group was calculated in relation to the initial serum glucose level as follows:

$$\% \text{ Serum glucose reduction} = [(C_0 - C_{24}/C_0)] \times 100$$

where C_0 is the mean initial fasting serum glucose level, C_{24} is the mean serum glucose level 24 hours after treatment.

Table S1. Molecular formulae, molecular weights and elemental analyses data of compounds **7a-e** and **8a-e**.

Comp. No.	Mol. Formula (Mol. Wt.)	Analysis: % Calcd. (Found)			
		C	H	N	S
**7a**	$C_{22}H_{30}N_2OS$ (370.55)	71.31 (71.18)	8.16 (8.24)	7.56 (7.42)	8.65 (8.65)
**7b**	$C_{22}H_{29}ClN_2OS$ (405.0)	65.24 (65.11)	7.22 (7.34)	6.92 (6.90)	7.92 (7.90)
**7c**	$C_{22}H_{29}BrN_2OS$ (449.45)	58.79 (58.65)	6.50 (6.52)	6.23 (6.21)	7.13 (7.22)
**7d**	$C_{22}H_{29}N_3O_3S$ (415.55)	63.59 (63.24)	7.03 (7.22)	10.11 (9.98)	7.72 (7.68)
**7e**	$C_{24}H_{28}F_6N_2OS$ (506.55)	56.91 (56.62)	5.57 (5.65)	5.53 (5.50)	6.33 (6.35)
**8a**	$C_{28}H_{35}N_3S$ (445.66)	75.46 (75.38)	7.92 (8.00)	9.43 (9.42)	7.19 (7.21)
**8b**	$C_{28}H_{34}ClN_3S$ (480.11)	70.05 (69.88)	7.14 (7.22)	8.75 (8.72)	6.68 (6.67)
**8c**	$C_{28}H_{34}BrN_3S$ (524.56)	64.11 (64.09)	6.53 (6.64)	8.01 (7.96)	6.11 (6.13)
**8d**	$C_{28}H_{34}N_4O_2S$ (490.66)	68.54 (68.54)	6.98 (7.01)	11.42 (11.35)	6.54 (6.51)
**8e**	$C_{30}H_{33}F_6N_3S$ (581.66)	61.95 (61.90)	5.72 (5.76)	7.22 (7.05)	5.51 (5.53)
